# Ultrasound-assisted deep eutectic solvent extraction of bioactive flavonoids from *Anchusa italica* retz.: optimization, characterization, and evaluation of antidepressant potential − targeting oxidative stress and neurotrophic pathways

**DOI:** 10.1016/j.ultsonch.2025.107598

**Published:** 2025-10-04

**Authors:** Bingchen Han, Qindan Cui, Zhiliang Ma, Leiling Shi, Yu Sun, Jiawei Dai, Jun Deng, Han Cheng, Jun Li, Yuebin Ge, Xianju Huang

**Affiliations:** a Hubei International Science and Technology Cooperation Base (SH2311), South-Central Minzu University, Wuhan 430079, China; b School of Pharmaceutical Sciences, South-Central Minzu University, Wuhan 430079, China; c College of Life Sciences, South-Central Minzu University, Wuhan 430079, China; d Department of Pharmacy, Sichuan Provincial People’s Hospital East Sichuan & Dazhou First People’s Hospital, Dazhou 635000, China; e Qinghai Tibetan Medicine Research Institute, Xining, Qinghai 810016, China; f Xinjiang Institute of Chinese and Ethnic Medicine, Urumqi 830002, China

**Keywords:** *Anchusa italica* retz, Total flavonoids, Ultrasound assisted DES extraction, Antidepressant effects, Oxidative stress

## Abstract

This study optimized the extraction of flavonoids from *Anchusa italica* Retz. using ultrasound-assisted deep eutectic solvent (DES) technology to maximize yield. A Box–Behnken design identified the optimal extraction parameters: solid–liquid ratio of 32.97, temperature of 68.74 °C, extraction time of 101 min, and ultrasonic power of 480.68 W. Flavonoid components were identified by liquid chromatography–mass spectrometry and quantified using high-performance liquid chromatography. *In vitro*, the total flavonoid extract significantly reduced oxidative stress markers—malondialdehyde, reactive oxygen species, and iron—and enhanced antioxidant defense markers, including superoxide dismutase, glutathione, glutathione peroxidase, and brain-derived neurotrophic factor (BDNF) in lipopolysaccharide-stimulated BV2 microglia. These protective effects were potentially mediated through the Toll-like receptor 4/nuclear factor kappa B/glutathione peroxidase 4 (TLR4/NF-κB/GPX4) signaling pathway. *In vivo*, oral administration of the extract for four weeks ameliorated depression-like behaviors in C57BL/6 mice exposed to chronic unpredictable mild stress. Treatment with the extract elevated serum levels of the neurotransmitters dopamine and serotonin, as well as those of BDNF, while upregulating hippocampal protein expression in the BDNF/tyrosine receptor kinase B/cAMP response element-binding protein (BDNF/TrkB/CREB) signaling pathway, thereby confirming its antidepressant efficacy. This study establishes an efficient DES-based extraction method for bioactive flavonoids and provides compelling evidence for the therapeutic potential of *A. italica* flavonoids in treating depression by targeting oxidative stress and neurotrophic signaling.

## Graphical abstract

**Figure:**
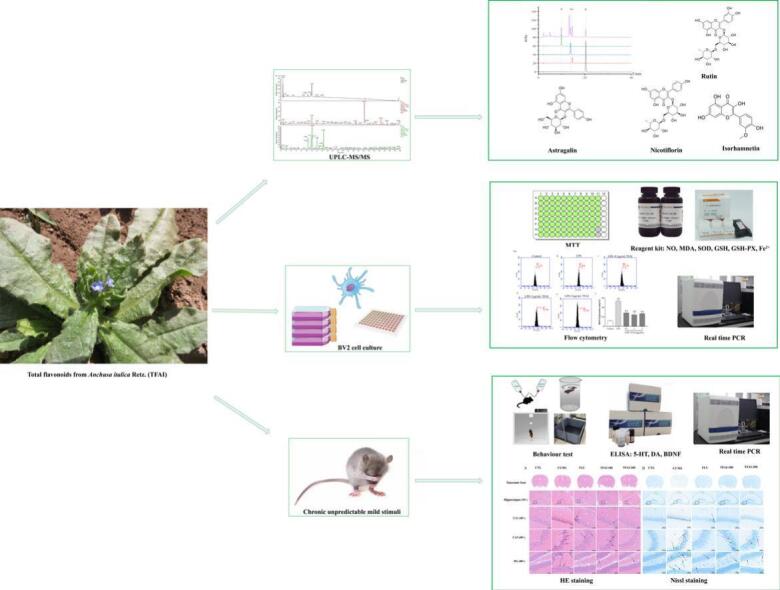

## 1 Introduction

*Anchusa italica* Retz.—locally known as “Gav zaban” in Persian—is a notable species within the Boraginaceae family [1], predominantly distributed across China, Syria, Iran, Afghanistan, and other regions. Traditionally, Kurdish communities have utilized this plant in culinary preparations [[2], [3], [4]], whereas in Uyghur medicine, it is considered a primary herbal remedy for cardiovascular and cerebrovascular conditions. Flowers of *A. italica* are traditionally used as a pediatric tonic and as antitussive, purgative, diuretic, and anti-inflammatory agents [5]. These therapeutic effects are attributed to a range of bioactive phytochemicals, including polyphenols, flavonoids, tannins, and terpenes [1,6].

Depression is among the most prevalent and complex mental health disorders, significantly impairing social functioning, occupational performance, and overall health. Although several pharmacological treatments are available—such as tricyclic antidepressants, selective dopamine reuptake inhibitors, selective norepinephrine reuptake inhibitors, and selective serotonin reuptake inhibitors—these are often associated with delayed therapeutic effects and undesirable side effects during long-term use [7,8]. Recent evidence highlights the role of dietary factors in modulating depression risk, suggesting that specific nutrients may influence its onset and progression [9].

Over recent decades, extensive research has investigated the antidepressant potential of natural compounds, particularly flavonoids [10]. Flavonoids exhibit diverse neuromorphological effects and, unlike synthetic antidepressants, often act on multiple molecular targets. Their antidepressant activity is attributed to modulation of various neuronal transmissions or pathways—such as the noradrenergic, serotonergic, GABAergic, and dopaminergic pathways—as well as inhibition of monoamine oxidase and tropomyosin receptor kinase B (TrkB) [11]. Additionally, flavonoids promote neurogenesis and upregulate brain-derived neurotrophic factor (BDNF) [12]. Nearly all fruits, grains, and vegetables are rich sources of flavonoids and possess the ability to prevent or reverse stress through multiple mechanisms [13].

Conventional methods for extracting flavonoids from plants are limited by low yields, high consumption of organic solvents, resource wastage, and environmental pollution [14]. Ultrasound-assisted extraction (UAE) has emerged as a promising alternative, utilizing acoustic cavitation—where ultrasound waves generate collapsing microbubbles—to enhance cell disruption and release of intracellular components [15]. Simultaneously, novel green solvents, especially deep eutectic solvents (DES), have gained attention due to their ease of synthesis, low cost, eco-friendliness, low volatility, high solvating capacity, and recyclability [16]. Recently, the integration of DES with UAE has emerged as a synergistic approach, providing a new paradigm for flavonoid extraction.

Total flavonoids from *A. italica* Retz. (TFAI)—the principal bioactive constituents of this traditionally used medicinal and edible plant—exhibit remarkable biological activities, including strong antibacterial and antioxidant effects, as well as cardioprotective properties against chronic myocardial infarction [3,17,18]. However, conventional extraction methods for TFAI are hindered by several limitations, such as low yield, high production costs, and thermal degradation of active components due to high-temperature processing. Additionally, the widely used hot water extraction method introduces considerable polysaccharide impurities, complicating downstream purification.

Notably, compared to flavonoid extracts from other plant sources, TFAI offer clear benefits. *A. italica* Retz.—a plant with a long history of safe application in both medicinal and dietary contexts—possesses distinct advantages: its long-term use in traditional practices implies favorable safety profiles; additionally, its biological activity is notably stronger. Despite these benefits, the potential antidepressant effects of TFAI remain largely unexplored, highlighting a critical gap in current research and an opportunity to further harness this valuable natural resource.

This study aimed to address two critical research gaps: (1) the limitations of conventional antidepressant therapies—such as adverse side effects and delayed onset of action—and those of traditional flavonoid extraction methods, which suffer from low yield and environmental impact; and (2) the underexplored potential of *A. italica* Retz. flavonoids in the management of depression. Accordingly, the primary objectives of this study were: (1) to establish an efficient and environmentally sustainable extraction method for TFAI using a synergistic ultrasound-assisted DES approach, and (2) to investigate the antidepressant potential of the optimized TFAI extract as a novel, multi-target dietary supplement, leveraging its intrinsic bioactivities and the growing evidence linking nutrition to mental health.

## 2 Materials and methods

### 2.1 Materials

*Anchusa italica* plants were purchased from Hainan (Lot no.: C30273307). Choline chloride (ChCl), urea (Urea), glucose (Glu), lactic acid (LA), oxalic acid (OA), malonic acid (MA), 1,4-butanediol (BDO), glycerol (Gly), and ethylene glycol (EG) were purchased from Sinopharm Chemical Reagent Co., Ltd. NaNO_2_, Al(NO_3_)_3_·9H_2_O, and NaOH were purchased from Shanghai Macklin Biochemical Technology Co., Ltd.

The murine microglial BV2 cell line was acquired from the China Center for Type Culture Collection (CCTCC, Wuhan University). Cells were cultured in DMEM (Gibco, USA; #8123208) supplemented with 10 % fetal bovine serum (Hangzhou Tianhang Biotechnology, China; #11011-8611) and 1 % penicillin–streptomycin (Gibco, USA; #2585654), and maintained at 37 °C in a 5 % CO_2_ atmosphere. Reagents used included lipopolysaccharide (LPS; Sigma-Aldrich, China; #L8274) and MTT (Ranjco Technology, China; #BS186). Assay kits GSH (A-006-2-1), GSH-Px (A-005-1-2), MDA (A-003-1-2), and SOD (A-001-3-2) were purchased from Nanjing Jiancheng Bioengineering Institute, China. BDNF (YJ002219), 5-hydroxytryptamine (5-HT; YJ001891), and dopamine (DA; YJ002024) were purchased from Shanghai Yiji Biotechnology Co., Ltd. (China). Anti-MEK1 Rabbit pAb (GB11304) and Anti-Phospho-CREB Rabbit pAb (GB114322) were purchased from Service Biotechnology Co., Ltd. (Wuhan, China).

### 2.2 Preparation of DESs

Accurately weigh each component according to the following molar ratios: Choline Chloride (ChCl) / Urea (Urea) 1:2, ChCl / Glucose (Glu) 1:1, ChCl / Lactic Acid (LA) 1:2, ChCl / Oxalic Acid (OA) 1:1, ChCl / Malonaldehyde (MA) 1:1, ChCl / 1,4-Butanediol (BDO) 1:5, ChCl / Glycerol (Gly) 1:2, and ChCl / Ethylene Glycol (EG) 1:1.See Table S1 for details. Each mixture was placed in a separate 50 mL beaker and labeled DES-1 through DES-8, respectively. The mixtures were stirred at 5000 rpm for 1 h at 80 °C using a DF-101S constant-temperature magnetic stirrer (Zhengzhou Kete Experimental Equipment Co., Ltd.) until homogeneous transparent solutions were obtained. The resulting DESs were cooled to room temperature and stored for later use.

### 2.3 Ultrasonic DES extraction of flavonoids from *A. italica* retz.: single-factor analysis

Dried *A. italica* samples were ground and sieved through a 70-mesh screen. Initial DES screening used a liquid-to-solid ratio of 1:30, extraction temperature of 50 °C, extraction time of 60 min, and ultrasonic power of 400 W. For optimal DES-6, the water content (0–50 %) was tested under these baseline conditions. Subsequent optimizations employed DES-6 with fixed parameters (50 °C, 60 min, and 400 W, unless varied): (1) Liquid-to-solid ratio (10:1–-50:1); (2) Extraction temperature (30–70 °C) at 1:30 ratio; (3) Extraction time (30–120 min) at 50 °C and 1:30 ratio; (4) Ultrasonic power (200–600 W) at 50 °C and 1:30 ratio. All experiments were performed in triplicate.

### 2.4 Optimization of extraction parameters for flavonoids from *A. italica* retz. using response surface methodology

Based on the results of single-factor experiments, four variables were selected for further optimization: liquid-to-solid ratio (A), extraction temperature (B), ultrasonic time (C), and ultrasonic power (D). Each variable was evaluated at three levels: Liquid-to-solid ratio (mL/g): 20, 30, and 40; Temperature (°C): 50, 60, and 70; Ultrasonic time (min): 80, 100, and 120; and Ultrasonic power (W): 400, 500, and 600. The total flavonoid yield was used as the response variable. A Box–Behnken design (BBD) was employed to construct the experimental matrix, and the data were analyzed using the Design-Expert software (version 8.0.6).

### 2.5 Determination of total flavonoid content

The total flavonoid content was determined using the NaNO_2_-Al(NO_3_)_3_-NaOH chromogenic system as described earlier [15]. Absorbance was measured at 510 nm using an ultraviolet–visible spectrophotometer. A standard calibration curve was constructed using rutin as the reference compound (y = 1.3881x + 0.0183; R^2^ = 0.9979), with a linear detection range of 0.0039 to 0.25 mg/mL. The total flavonoid content (Y) was calculated using the following equation:

(1) $$Y=\frac{C\times V\times N}{m}$$

where “Y” denotes the flavonoid yield (mg/g), “C” represents the concentration of the total flavonoid measured (mg/mL), “V” denotes the solution volume (mL), “N” denotes the dilution factor, and “m” represents the raw material mass (g).

### 2.6 Flavonoid purification

The extract was filtered and concentrated using a rotary evaporator. Then, it was subjected to gradient elution through a D101 macroporous resin. Initially, the column was eluted with water (ethanol:water; 0:100) for five column volumes, and the eluate was discarded. Subsequently, the elution solvent was switched to absolute ethanol (ethanol:water; 100:0) for another five column volumes. The resulting eluate was collected, concentrated by rotary evaporation under reduced pressure, and dried under vacuum at 45 °C to obtain TFAI. Each purification step was performed in triplicate to ensure reproducibility.

### 2.7 Analysis of chemical composition of TFAI using UPLC-Q-Exactive-MS/MS

Chemical composition of TFAI was analyzed using a Thermo Scientific Q Exactive coupled with an Ultimate 3000 UPLC system. Separation was carried out on an Accucore aQ column (100 × 2.1 mm; 2.6 μm). The mobile phase consisted of solvent A (acetonitrile) and solvent B (0.1 % formic acid in water), with a flow rate of 0.2 mL/min and the column temperature maintained at 25 °C. The gradient elution program was set as follows:0–3 min, 5 % A;3–7 min, 5 %–10 % A;7–9 min, 10 %–17 % A; 9–11 min, 17 %–20 % A;11–14 min, 20 %–24 % A;14–16 min, 24 %–28 % A;16–19 min, 28 %–30 % A;19–21 min, 30 %–35 % A;21–29 min, 35 %–37 % A;29–33 min, 37 %–55 % A;33–43 min, 55 %–63 % A;43–43.1 min, 63 %–95 % A;43.1–50 min, 95 %–5% A. The ion source was a heated electric spray ion source with both positive and negative ion detection modes. The parameters were as follows: spray voltage, 3200 V; capillary temperature, 300.00 °C, sheath gas volume flow rate, 40.00 Arb; auxiliary gas volume flow rate, 10.00 Arb; maximum injection current, 100.00 µA; probe heater temperature, 320.00 °C; scanning range, *m*/*z* 80–1200; and scanning mode, simultaneous acquisition of positive and negative ions. Data analysis was performed using Xcalibur 4.0 software for accurate mass determination, fitting molecular formulae and compound identification through mzCloud database matching.

### 2.8 Determination of major compounds in TFAI using HPLC-DAD

HPLC-DAD was employed to identify the major constituents of TFAI. A C18 column (250 × 4.6 mm; 5 μm, Agilent), with a 10 μL injection volume and a flow rate of 1 mL/min, was used. Two solvents—A (0.4 % aqueous phosphate) and B (methanol)—were used for the gradient elution at 25 °C. The gradient program was set for 0–40 min, with a linear change in the mobile phase composition from A/B (65/35, v/v) to A/B (35/65, v/v). Detection was performed at 254 nm and each sample was analyzed in triplicate.

### 2.9 Cellular experiments

#### 2.9.1 Cell viability assay

BV2 microglial cells were seeded in a 96-well plate and pretreated with TFAI (0.5, 1, or 5 μg/mL) for 3 h, followed by co-exposure to LPS (5 μg/mL) for 24 h. Cell viability was assessed using 3-(4,5-dimethylthiazolyl-2-yl)-2,5-diphenyl tetrazolium bromide (MTT) assay, with absorbance measured at 570 nm.

#### 2.9.2 Griess assay

To determine the optimal LPS concentration for induction of inflammation, BV2 cells (1 × 10^4^ cells/well in 96-well plates) were treated with LPS (0.5, 1, 5, 10, 25, or 50 μg/mL) for 24 h. NO production (measured as nitrite in the supernatant using the commercial NO assay kit) was assayed, identifying 5 μg/mL as the optimal LPS concentration. To evaluate the optimal TFAI treatment concentration, cells were pretreated with TFAI (0.5, 1, 5, 10, 50, or 100 μg/mL) for 3 h, followed by stimulation with 5 μg/mL LPS. After 24 h, NO production was measured using the same kit.

#### 2.9.3 BDNF, MDA, SOD, GSH, GSH-Px assays

BV2 cells were pretreated with TFAI (0.5, 1, or 5 μg/mL) for 3 h, followed by co-incubation with LPS (5 μg/mL) for 24 h. Cell supernatants were collected for quantification of BDNF using an ELISA kit, following the manufacturer's protocol. Simultaneously, intracellular levels of oxidative stress markers—MDA, SOD, GSH, and GSH-Px—were quantified using corresponding commercial kits.

#### 2.9.4 Lipid reactive oxygen species (ROS) and iron ion content detection

BV2 cells (2 × 10^5^ cells/well in 6-well plates) were pretreated with TFAI (0.5, 1, or 5 μg/mL) for 3 h followed by co-exposure with LPS (5 μg/mL) for 24 h. After washing three times with DMEM, cells were incubated with 10 μM DCFH-DA at 37 °C for 30 min. ROS levels were quantified by flow cytometry.

### 2.10 Animal experiments

#### 2.10.1 Animal model and chronic unpredictable mild stress (CUMS) protocol

Male C57BL/6 mice (6–8 weeks old; 18–20 g) were housed under controlled conditions (22 ± 2 °C, 12 h light/dark cycle). Following acclimatization, mice were randomly assigned to either the control group (n = 10, no stress) or CUMS groups (n = 59). The CUMS protocol, consisting of variable daily stressors (2–3 types/day) in a non-repetitive sequence (Table S2), was applied for 9 weeks. At week 5, the sucrose preference tests identified depressed mice; non-responders were excluded. The remaining CUMS mice (n = 40) were subdivided into four groups (n = 10/group): CUMS (saline), CUMS + Flu (fluoxetine; 20 mg/kg), CUMS + TFAIL (TFAI low dose; 100 mg/kg), and CUMS + TFAIH (TFAI high dose; 200 mg/kg). Beginning from week 6, treatments (saline, fluoxetine, or TFAI in saline) were orally administered once daily (9:00–11:00 am) for 4 weeks. All procedures complied with institutional ethical guidelines (South-Central Minzu University, Approval No. 2021-scuec-050).

#### 2.10.2 Behavioral assessments (TST, SPT, FST, OFT)

Behavioral tests were conducted in a quiet environment. For TST, mice were suspended by the tail for 6 min, with immobility time recorded during the final 4 min. The SPT was conducted twice: at week 5 (pre-treatment) and at week 9 (pre-sacrifice), following 12 h of food and water deprivation. Mice were individually housed and allowed simultaneous access to water and 1 % sucrose for 24 h (bottle positions swapped at 12 h). Sucrose preference (%) was calculated as follows: Sucrose preference = (sucrose intake / total fluid intake) × 100. The FST was performed at week 9 by placing mice in 25 ± 1 °C water for 6 min; immobility was recorded during the last 4 min. The OFT, also conducted at week 9, recorded the number of crossings during the final 4 min of a 6-min session conducted in a gray arena. All equipment was cleaned with 75 % ethanol between trials.

#### 2.10.3 Molecular and histological analyses

Total RNA was extracted from hippocampal tissues and BV2 cells, and reverse-transcribed to cDNA. qRT-PCR was performed using SYBR Green chemistry under the following conditions: 95 °C for 3 min, followed by 40 cycles of 95 °C for 5 s and 60 °C for 34 s. Gene expression levels were normalized to GAPDH using the 2^−ΔΔct^ method. Primer sequences are listed in Table S3.

Mice were euthanized via cervical dislocation. Organ indices (liver, spleen, brain, and thymus) were calculated as follows: organ index = (organ weight/body weight) × 100 %. Hippocampal tissues were either flash-frozen for molecular analysis or fixed in formalin for histological examination. Formalin-fixed brain sections were stained with hematoxylin and eosin (HE) and Nissl stain to assess neuronal integrity in hippocampal subregions (CA1, CA3, and DG).

Serum and hippocampal tissue samples were collected from CUMS-exposed mice, and protein concentrations were quantified using a BCA protein assay kit. Levels of BDNF, 5-HT, and DA in serum, as well as BDNF in hippocampal tissue, were measured using commercially available kits, according to the manufacturers’ instructions.

For immunohistochemistry, paraffin sections were incubated overnight at 4 °C with primary antibodies against MEK1 (1:100) and p-CREB (1:100), followed by incubation with appropriate secondary antibodies and DAB staining. Positive staining was quantified as integrated optical density using IPP 6.0 software across five randomly selected fields per sample.

### 2.11 Statistical analyses

Data are presented as mean ± SEM. GraphPad Prism 9 software (GraphPad Software Inc., San Diego, CA, USA) was used for generating graphs. A one-way analysis of variance (ANOVA) followed by Tukey’s post hoc test was performed for group comparisons. Statistical significance was set at *p* < 0.05.

## 3 Results

### 3.1 Extraction and identification of components in total flavonoids from *A. italica* retz

**Fig. 1:**
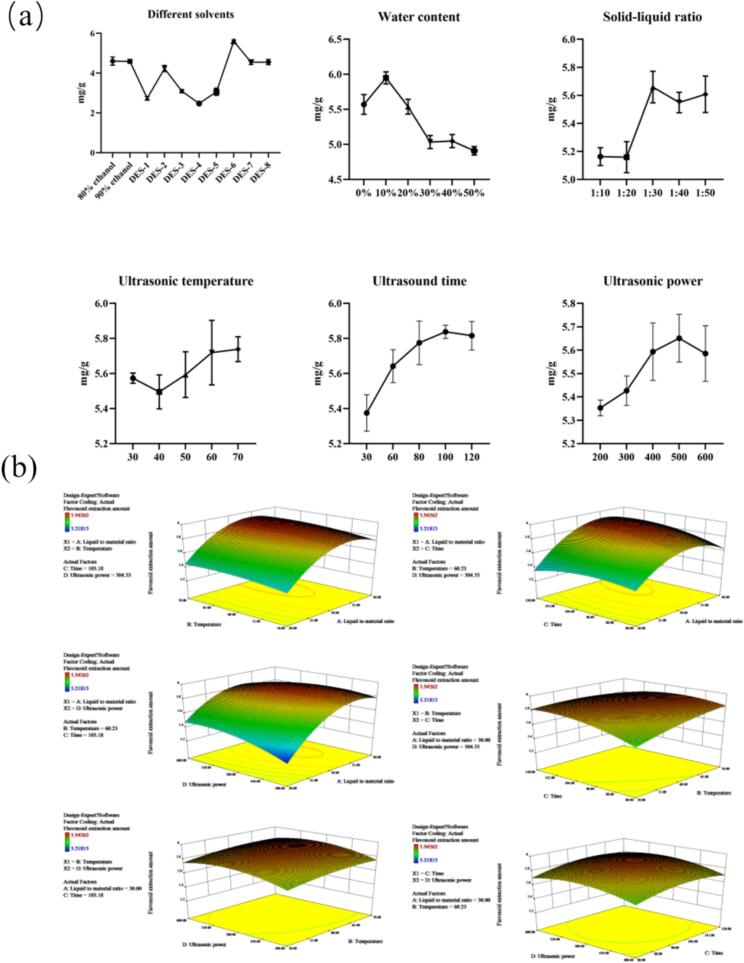
(a) Results of Single-Factor Experiment on Flavonoid Extraction from *Anchusa italica* Retz.; (b) Response surface plots of the model for extraction yield of DES-6. Each experimental sample was designed with three parallel replicates.

The optimal extraction solvent—DES-6—was identified via single-factor experiments based on DES type and DES water content. The results showed that DES-6 exhibited significantly higher extraction efficiency compared to 80 % ethanol, with a 21.59 % increase in flavonoid yield. Among all conditions tested, 10 % water content was found to be optimal for extraction. Therefore, DES-6 with 10 % water content was selected for flavonoid extraction, and the process was optimized. Additionally, single-factor experiments investigating solid–liquid ratio, temperature, ultrasonic time, and ultrasonic power were used to define parameter ranges for subsequent response surface design. The results of these experiments are presented in Fig. 1a.

Finally, a four-factor, three-level response surface design was implemented with the following variables: (A) solid–liquid ratio (30, 40, or 50 mg/g), (B) temperature (50, 60, or 70 °C), (C) ultrasonic time (80, 100, or 120 min), and (D) ultrasonic power (400, 500, or 600 W). The experimental schemes and results are summarized in Table 1. A quadratic polynomial regression equation (Formula 2) was used to describe the relationship between the response variable and independent variables. The ANOVA results of the response surface analysis (Table S4) showed that the fitted model had a Prob > F value < 0.05, indicating that the regression model was highly significant. The lack-of-fit term was not significant (*p* > 0.05), indicating a good model fit and validating the applicability of the response surface model for further optimization. Notably, the interaction term AD and quadratic terms A^2^, D^2^ had Prob > F values < 0.01, indicating extremely significant effects on flavonoid extraction yield. The relative influence of each factor on extraction efficiency was ranked as follows: solid–liquid ratio > ultrasonic power > ultrasonic time > temperature.

**Table 1:** Results of extraction yield for flavonoids from *Anchusa italica* retz. using response surface methodology design with ultrasound-assisted extraction.

Run	A:Liquid to material ratio (v/m)	Temperature (°C)	Time (min)	Ultrasonic power (W)	Flavonoid extraction amount (mg/g)
1	20	60	80	500	5.448
2	30	70	100	400	5.732
3	20	60	100	400	5.218
4	40	70	100	500	5.837
5	30	60	80	600	5.702
6	30	60	100	500	5.852
7	40	60	120	500	5.824
8	30	50	100	400	5.727
9	40	50	100	500	5.745
10	30	70	100	600	5.794
11	30	60	100	500	5.898
12	30	50	100	600	5.785
13	30	70	120	500	5.845
14	20	50	100	500	5.380
15	20	70	100	500	5.439
16	30	50	80	500	5.535
17	40	60	100	400	5.761
18	40	60	100	600	5.701
19	30	70	80	500	5.870
20	20	60	120	500	5.383
21	30	60	120	400	5.704
22	40	60	80	500	5.617
23	20	60	100	600	5.468
24	30	60	100	500	5.943
25	30	60	100	500	5.852
26	30	50	120	500	5.768
27	30	60	100	500	5.833
28	30	60	120	600	5.771
29	30	60	80	400	5.661

Flavonoid extraction amount = 5.88 + 0.18 × A + 0.048 × B + 0.039 × C + 0.035 × D + 8.288 × 10^-3^ × A × B + 0.068 × A × C − 0.077 × A × D × 0.064 × B × C + 9.978 × 10^-4^ × B × D + 6.501 × 10^-3^ × C × D − 0.24 × A^2^ − 0.035 × B^2^ − 0.077 × C^2^ − 0.090 × D^2^（Formula 2）.

Response surface plots illustrating the interactions among extraction parameters affecting total flavonoid extraction were generated using the Design-Expert 8.0.6 software (Fig. 1b). Predictive analysis identified the optimal UAE conditions as follows: solid–liquid ratio, 32.97; temperature, 68.74 °C; extraction time, 101 min; and ultrasonic power, 480.68 W. Under these conditions the predicted yield was 5.919 mg/g, closely matching the experimentally obtained yield of 5.938 mg/g, thereby confirming the accuracy and reliability of the response surface-optimized process.

In scale-up experiments using 500 g of *A. italica*, the total flavonoid yield reached 6.193 mg/g. The extract was further purified using D101 macroporous resin, resulting in a TFAI dry powder with a yield of 2.2 % and a total flavonoid content of 27.72 %.

**Fig. 2:**
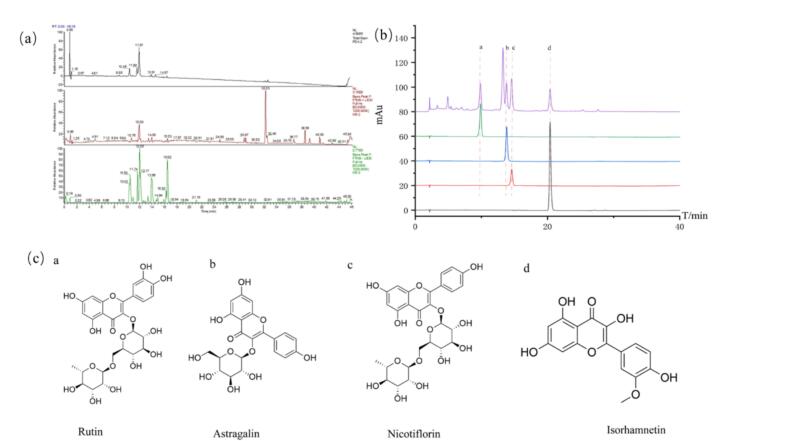
Results of TFAI chemical composition analysis. (a) Total ion flow diagram of TFAI, top: PDA diagram, middle: positive ion mode, bottom: negative ion mode; (b) High performance liquid chromatography of four components in TFAI; a:Rutin, b: Astragalin, c: Nicotiflorin, d: Isorhamnetin. (c) Chemical structures of 4 compounds in TFAI.Each experimental sample was designed with three parallel replicates.

The UPLC-Q-Exactive-MS/MS technique was used for component profiling of TFAI under the above mentioned chromatographic and mass spectrometric conditions. Data were acquired in both positive and negative ionization modes, and total ion flow diagrams were generated for each mode (Fig. 2a). The Xcalibur 4.0 software was used to analyze and identify the compounds in conjunction with the Oxalis autochthonous database, the mzCloud database, and literature references. A total of 17 compounds were identified, including nine flavonoids (for example, Nicotiflorin, Rutin, Kaempferol, Isorhamnetin, Quercetin, Astragalin) and two organic acids (Pinolenic acid and Caffeic acid). Detailed results are provided in Supplementary Table S5. Peak area ratios confirmed that flavonoids were the predominant components of the extract. Quantitative analysis of the four major flavonoids—Astragalin, Isorhamnetin, Rutin, and Nicotiflorin—was performed using HPLC (Fig. 2 and c), revealing their respective contents in TFAI as 1.425 %, 0.387 %, 1.130 %, and 1.479 %. The standard curves, linearity ranges, and content data for these compounds are summarized in Supplementary Material Table S6.

### 3.2 Effect of TFAI on LPS-induced inflammation in BV2 cells

**Fig. 3:**
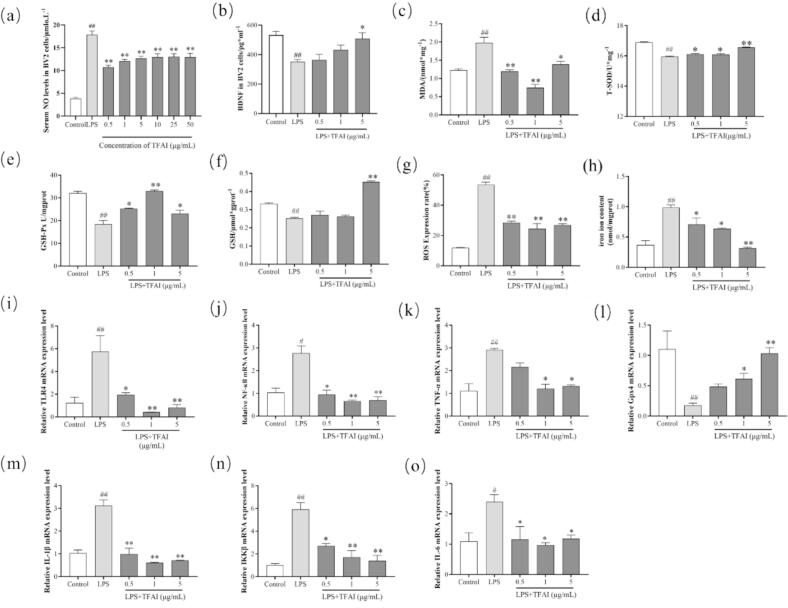
(a) TFAI pretreated cells BV2 cells for 3 h then LPS co-treated for 24 h post cellular supernatant NO content; (b) Effect of TFAI on BV2 cells of BDNF; (c) Effect of TFAI on intracellular MDA; (d) Effect of TFAI on intracellular T-SOD; (e) Effect of TFAI on intracellular GSH-PX; (f) Effect of TFAI on intracellular GSH-PX in BV2 cells; (g) Fluorescence intensity analysis of ROS; (h) Effect of TFAI on intracellular Iron ion content in BV2 cells; (i) Relative expression of TLR4 mRNA; (j) Relative expression of NF-κB mRNA; (k) Relative expression of TNF-α mRNA; (l) Relative expression of Gpx4 mRNA; (m) Relative expression of IL-1β mRNA; (n) Relative expression of IKKβ mRNA; (o) Relative expression of IL-6 mRNA; BDNF: Brain-Derived Neurotrophic Factor; MDA: Malondialdehyde; T-SOD: Total Superoxide Dismutase; GSH: Glutathione; GSH-PX: Glutathione peroxidase; ROS: Reactive Oxygen Species; ^##^*P < 0.01*, ^#^*P < 0.05* versus Control; ^**^P < 0.01, *P < 0.05 versus LPS-injured group; n = 5:Five biological replicates per experimental group.

The results of the cellular model construction are shown in Fig. S1. TFAI reduced BV2 cell viability with an IC_50_ of 296.5 µg/mL, with viability reaching its lowest point at 200 µg/mL (*p* < 0.01 vs. control). Concentrations below 100 µg/mL had no significant detrimental effect on cell viability and were therefore used in subsequent experiments. LPS (0.1–10 µg/mL) significantly increased NO production in BV2 cells, with 5 µg/mL chosen for stimulation. Pretreatment with TFAI (0.5–50 µg/mL) for 3 h significantly inhibited LPS-induced NO release (*p* < 0.01 vs. LPS alone; Fig. 3a).

TFAI exhibited notable neuroprotective effects in LPS-stimulated BV2 cells by modulating key oxidative stress markers (Fig. 3, Fig. 3). Compared to controls, LPS significantly decreased BDNF (*p* < 0.01) and GSH (*p* < 0.05) levels, while markedly increasing MDA (*p* < 0.01), ferric iron (*p* < 0.01), and ROS (*p* < 0.05) levels. TFAI treatment effectively reversed these LPS-induced alterations as follows: 5 μg/mL TFAI significantly elevated BDNF levels (*p* < 0.01); all tested concentrations of TFAI—0.5, 1, and 5 μg/mL—significantly decreased MDA (vs LPS; *p* < 0.05 to *p* < 0.01) and ROS (vs LPS; *p* < 0.01) levels; TFAI also significantly increased SOD and GSH-Px levels (vs LPS; *p* < 0.05 to *p* < 0.01); and reduced ferric iron accumulation in a dose-dependent manner (vs LPS; *p* < 0.05 to *p* < 0.01). Notably, only 5 μg/mL TFAI significantly restored GSH content (vs LPS; *p* < 0.01). Collectively, these results suggest that TFAI exert antidepressant effects by significantly enhancing BDNF, reducing ferric iron accumulation, and mitigating oxidative stress in LPS-induced inflammatory conditions.

GPX4 plays a critical role in preventing ferroptosis by reducing phospholipid hydroperoxides, thereby inhibiting lipid peroxidation. In lipid peroxidation-mediated diseases, GPX4 activation suppresses NF-κB signaling. In the present study, compared to that in the controls, mRNA expression levels of pro-inflammatory cytokines and signaling molecules—TLR4, IKKβ, NF-κB, IL-1β, and TNF-α—were significantly elevated in LPS treated cells (*p* < 0.05). Treatment with TFAI (1 or 5 μg/mL) significantly decreased the mRNA expression of these markers in BV2 cells compared to that in the LPS group (*p* < 0.05 and *p* < 0.01; Fig. 3, Fig. 3).

### 3.3 Effects of TFAI on the CUMS mouse model

**Fig. 4:**
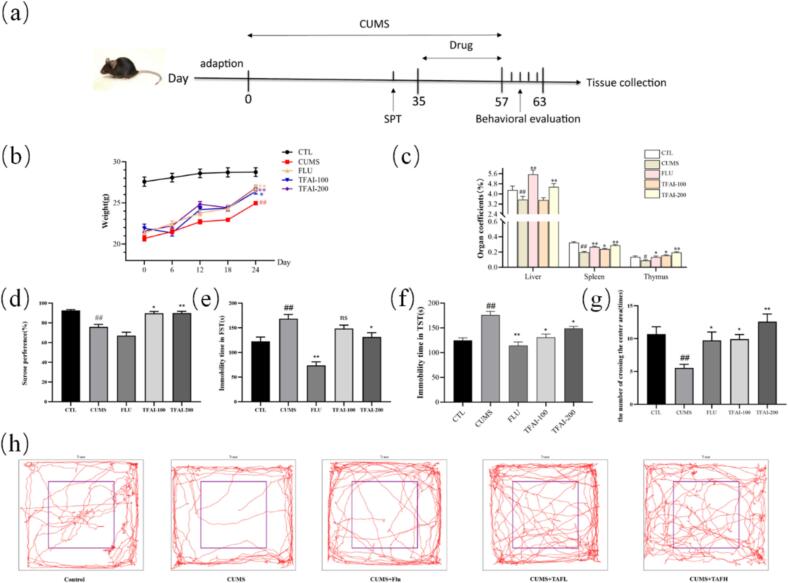
(a) Flow chart of *in vivo* experimental design; (b) Effect of TFAI on body weight changes in CUMS-exposed mice; (c) Effect of TFAI on liver, spleen and thymus organ indices in CUMS-exposed mice; (d) Effect of TFAI on sucrose preference in mice; (e) Effect of TFAI on immobilization time in force swimming test; (f) Effect of TFAI on immobilization time in tail suspension test; (g) Effect of TFAI on the number of crossings through the center region in open field test; (h) A figurative diagram of the trajectories of mice in the open field test; CTL: control group; FUL: fluoxetine; CUMS: chronic unpredictable mild stress; TFAI-100: 100 mg/kg total flavonoids from *Anchusa italica* Retz.; TFAI-200: 200 mg/kg total flavonoids from *Anchusa italica* Retz.; ^##^*P < 0.01*, ^#^*P < 0.05* versus CTL; ^**^P < 0.01, *P < 0.05 versus CUMS group; n = 10:Ten biological replicates per experimental group.

Body weight changes are commonly associated with depression, and depressive animal models typically exhibit weight loss. As shown in Fig. S2, control mice displayed steady weight gain over time, whereas CUMS exposure for 5 weeks caused significant weight loss (*p* < 0.01). After 4 weeks of treatment, mice in both fluoxetine and TFAI groups exhibited significantly higher body weights than those in the CUMS group (*p* < 0.05, *p* < 0.01; Fig. 4b). Anhedonia was assessed via SPT; mice in the CUMS group showed significantly reduced sucrose preference relative to controls (*p* < 0.01, Fig. S2), confirming anhedonia. Furthermore, CUMS significantly reduced all organ indices (liver/spleen/thymus) compared to the controls *p* < 0.01, *p* < 0.05; Fig. 4c). Mice in both fluoxetine and TFAI groups showed elevated organ indices, indicating reversal of CUMS-induced organ impairment.

The SPT results revealed that the sucrose preference rate of mice in the CUMS group was significantly lower than that of mice in the control group (*p* < 0.01; Fig. 4d), showing a depressive-like state. In contrast, mice in the TFAI group had a significantly higher preference rate than those in the CUMS group (*p* < 0.05 or *p* < 0.01), indicating TFAI's ability to improve the reward mechanism dysregulation. The FST results showed that CUMS mice exhibited significantly longer immobility durations than mice in the control group (*p* < 0.01; Fig. 4e), suggesting desperation. Mice treated with fluoxetine or TFAI showed significantly reduced immobility durations than those in the model group (*p* < 0.01 for fluoxetine and low-dose TFAI; *p* < 0.05 for high-dose TFAI), indicating improved depressive behavior. Similarly, TST results showed that CUMS mice had significantly prolonged immobility (*p* < 0.01; Fig. 4f.) Treatment with both fluoxetine and TFAI significantly shortened the immobility time (*p* < 0.01 for fluoxetine; *p* < 0.05 for low- and high-dose TFAI). The OFT results showed that CUMS mice entered the center area less often than mice in the control group (*p* < 0.01; Fig. 4, Fig. 4), consistent with increased anxiety. Treatment with fluoxetine and TFAI increased center crossings, with significant improvements observed in mice in the TFAI-treated groups (*p* < 0.05 for 100 mg/kg; *p* < 0.01 for 200 mg/kg).

**Fig. 5:**
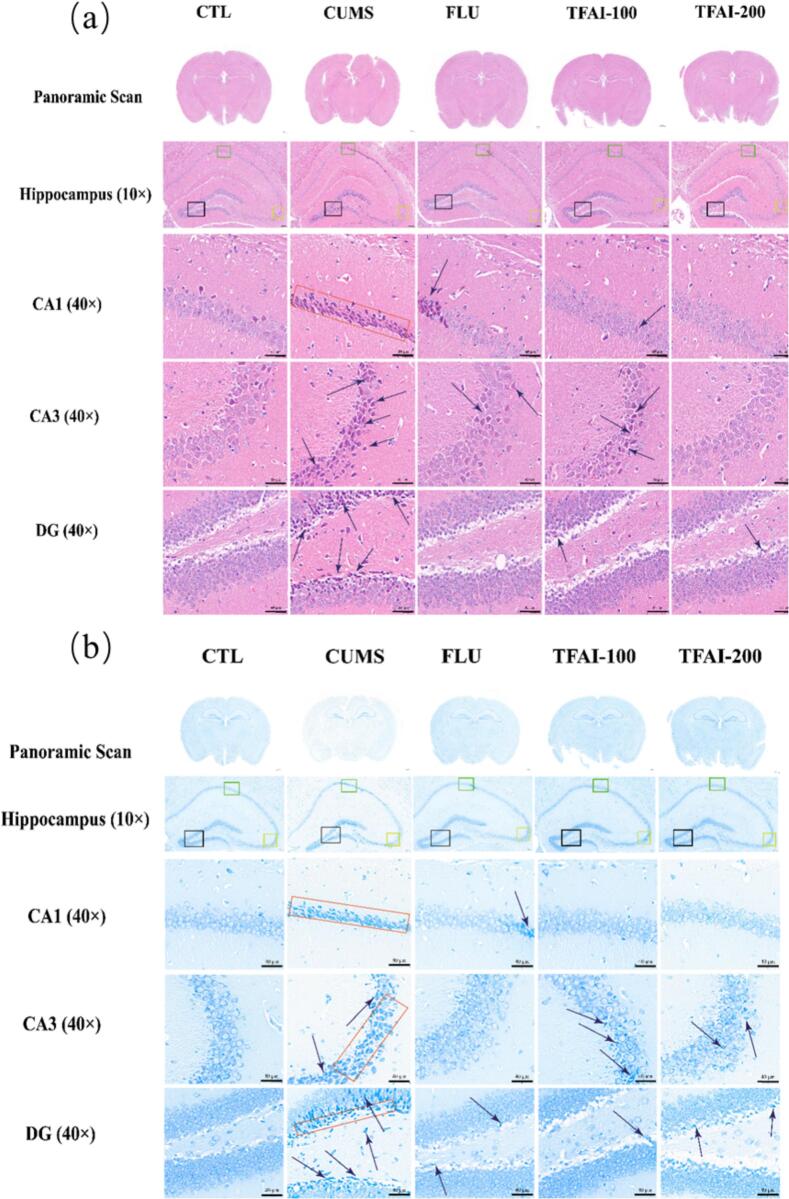
Effects of TFAI on CA1, CA3 and DG regions in mouse hippocampal tissue; (A) Effect of TFAI on HE staining of brain tissues in CUMS mice; (B) Effect of TFAI on Nissl staining of brain tissues in CUMS mice; CTL: control group; FUL: fluoxetine; CUMS: chronic unpredictable mild stress; TFAI-100: 100 mg/kg total flavonoids from *Anchusa italica* Retz.; TFAI-200: 200 mg/kg total flavonoids from *Anchusa italica* Retz.

The HE staining results showed that the hippocampal neurons of mice in the control group were abundant, closely arranged, and morphologically intact (Fig. 5a). In contrast, mice in the CUMS group exhibited a reduced number of neurons, a disordered and sparse arrangement, and atrophied, deformed cells with conical or polygonal shapes. TFAI treatment improved neuronal integrity, increased neuronal count, and restored orderly cellular arrangement and structure. Notably, the CA3 region of mice in the high-dose TFAI group demonstrated the most pronounced recovery, approaching normal levels; furthermore, the karyopyknosis in the CA1/DG regions was significantly alleviated.

Results of Nissl staining further supported these findings (Fig. 5b). Mice in the control group exhibited abundant Nissl bodies, indicative of active protein synthesis. In contrast, mice in the CUMS group showed reduced Nissl bodies and loosely arranged neurons, suggesting impaired protein synthesis. Treatment with TFAI and fluoxetine restored Nissl body density and neuronal organization, and partial restoration of protein synthesis. Collectively, these histological results demonstrate that TFAI mitigate CUMS-induced hippocampal neuronal damage and partially restore cellular functions.

**Fig. 6:**
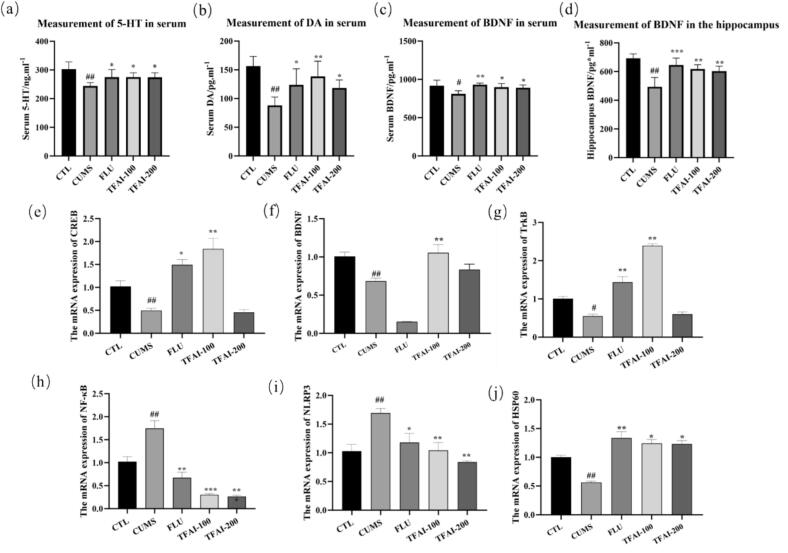
(a) The content of 5-HT in serum; (b) The content of DA in serum; (c) The content of BDNF in serum; (d) The content of BDNF in hippocampal tissue; CTL: control group; FUL: fluoxetine; CUMS: chronic unpredictable mild stress; (e) Relative expression of CREB mRNA; (f) Relative expression of BDNF mRNA; (g) Relative expression of TrkB mRNA; (h) Relative expression of NF-κB mRNA; (i) Relative expression of NLRP3 mRNA; (j) Relative expression of HSP60 mRNA; CTL: control group; FUL: fluoxetine; CUMS: chronic unpredictable mild stress; TFAI-100: 100 mg/kg total flavonoids from *Anchusa italica* Retz.; TFAI-200: 200 mg/kg total flavonoids from *Anchusa italica* Retz.; ^##^*P < 0.01*, ^#^*P < 0.05* versus CTL; ^**^P < 0.01, *P < 0.05 versus CUMS group; n = 5:Five biological replicates per experimental group.

Additionally, treatment with TFAI elevated the levels of BDNF, DA, and 5-HT in both the serum and hippocampal tissue of CUMS mice (Fig. 6, Fig. 6). Compared to controls, CUMS-exposed mice showed significantly reduced serum levels of 5-HT and DA (*p* < 0.01), and BDNF (*p* < 0.05), along with reduced hippocampal BDNF (*p* < 0.01). Fluoxetine (20 mg/kg) and TFAI (100 mg/kg or 200 mg/kg) treatment significantly reversed these reductions in serum (*p* < 0.05 or *p* < 0.01; 5-HT, DA, and BDNF) and hippocampal tissue (*p* < 0.01 or *p* < 0.001; BDNF) in treated mice compared to those in the CUMS model group.

The BDNF/TrkB/CREB pathway plays a critical role in synaptic plasticity. In the present study, CUMS mice showed significantly reduced hippocampal mRNA expression of BDNF, TrkB, and CREB compared to controls (*p* < 0.05 or *p* < 0.01; Fig. 6, Fig. 6). Mice in the TFAI-treated group showed significant upregulation in the expression of these genes relative to those in the CUMS group (*p* < 0.01). Conversely, CUMS significantly increased hippocampal expression of NF-κB and NLRP3—markers associate with neuroinflammation (*p* < 0.01 vs. control). TFAI administration significantly reduced the expression of both inflammatory markers in a dose dependent manner (*p* < 0.05 or *p* < 0.01), indicating anti-inflammatory effects.

**Fig. 7:**
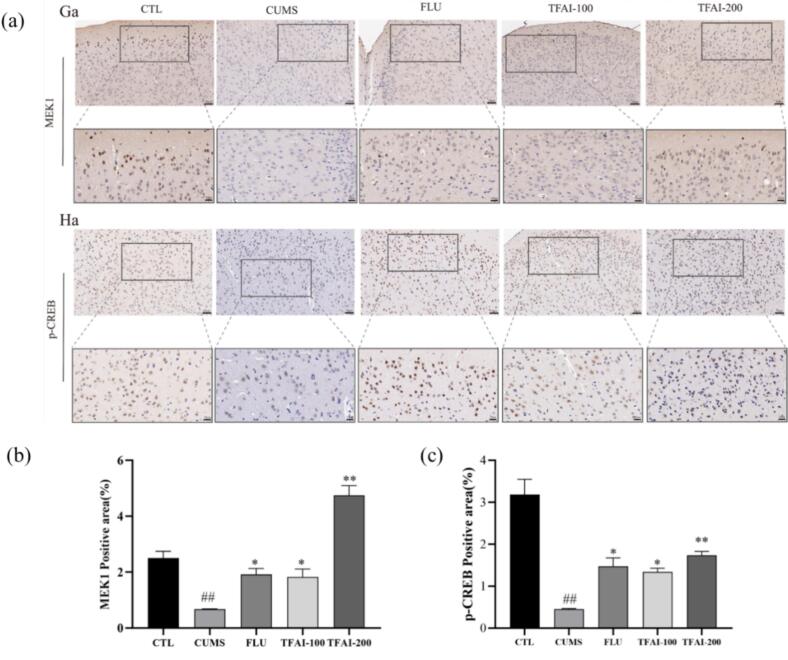
(a) Representative image of MEK1 and p-CREB immunohistochemical staining; (b) Quantification of MEK1-positive regions; (c) Quantification of p-CREB-positive regions; CTL: control group; FUL: fluoxetine; CUMS: chronic unpredictable mild stress; TFAI-100: 100 mg/kg total flavonoids from *Anchusa italica* Retz.; TFAI-200: 200 mg/kg total flavonoids from *Anchusa italica* Retz.; ^#^*P < 0.05* versus CTL; *P < 0.05, ^**^P < 0.01 versus CUMS group, n = 5:Five biological replicates per experimental group.).

Immunohistochemistry analysis revealed reduced MEK1 and p-CREB protein expression in CUMS mice (Fig. 7). Mice in both fluoxetine- and TFAI-treated groups showed significant increase in their expression compared to those in the CUMS group (*p* < 0.01), with mice in the high-dose TFAI group showing the greatest effect. Overall, these findings indicate that TFAI exert antidepressant effects by activating BDNF and suppressing the NF-κB/NLRP3-mediated inflammatory response.

## 4 Discussion

Numerous studies indicate that herbal dietary supplements may help prevent and alleviate depression, largely due to the presence of bioactive components such as flavonoids, polyphenols, and alkaloids [19]. *A. italica* Retz. has a long history of dietary use, employed by Kurds in the Middle East for food preparation and by the Uyghur ethnic group in China as an herbal remedy [3]. The plant offers various beneficial properties, particularly for cardiovascular health. However, despite its high flavonoid content, studies investigating it or its flavonoid constituents as potential dietary supplements for depression are limited. A key challenge in utilizing *A. italica* flavonoids is the low extraction efficiency associated with traditional solvent extraction methods.

To address this limitation, we developed a UAE method using a DES system. Among the tested solvents, ChCl/BDO—at a molar ratio of 1:5—was identified as the most efficient for flavonoid extraction. Extraction parameters were optimized using single-factor experiments and response surface methodology. BDO functions as a potent hydrogen bond donor (HBD) owing to its two hydroxyl groups (–OH) [20]. The high HBD ratio (ChCl:HBD = 1:5) ensures a substantial presence of free hydroxyl groups within the DES. Under ultrasonication, these free hydroxyl groups effectively disrupt plant cell walls and membranes and form strong hydrogen bonds with the target flavonoid molecules, thereby enhancing their release [21].

We characterized the chemical composition of TFAI and quantified key flavonoid constituents, thereby establishing a foundational dataset for its material basis. This optimized ultrasound-assisted DES extraction followed by macroporous resin enrichment enabled us to obtain 22 mg of extract per gram of dry *A. italica* Retz. material, with a flavonoid content of 27.72 %. This high-yield extract provides a robust foundation for evaluating the potential antidepressant properties of *A. italica* Retz. flavonoids.

The central nervous system is particularly vulnerable to oxidative stress due to its high oxygen consumption, limited antioxidant defenses, large membrane surface-to-cytoplasm ratio, and abundance of polyunsaturated fatty acids and metal ions [22]. Elevated levels of pro-inflammatory cytokines, including IL-1β, IL-6, TNF-α, and other acute-phase proteins, have been consistently reported in patients with depression [23,24]. Microglial cells, which perform essential neuronal and immune functions, typically exist in a resting state under physiological conditions. Upon activation by pro-inflammatory cytokines, they participate in neurotrophic activities such as synaptic remodeling, neurogenesis, and the release of neurotrophic factors [25]. Notably, TrkB agonists have been shown to mitigate inflammation in microglial cells and preserve the survival of neurons under degenerative conditions [26]. Furthermore, various antidepressants—regardless of their assumed specificity for monoamine targets—have been shown to reduce the release of inflammatory mediators in immune cells *in vitro* [27]. These findings collectively highlight the significant role of neuroinflammation in the pathophysiology of depression.

Excessive iron can be detrimental because abnormal accumulation of iron and ROS is associated with various pathologies, including iron overload disorders and cancer [28]. In neurodegenerative diseases, iron overload triggers the polarization of microglial cells into the pro-inflammatory (M1) phenotype, leading to elevated secretion of TNF-α and IL-1β, and thereby promoting neuroinflammation [29]. Disruptions in cysteine metabolism leads to a reduction in GSH levels, impairing the activity of GPX4 [30]. GPX4 is a key downstream regulator of ferroptosis, catalyzing the GSH-dependent reduction of lipid hydroperoxides to non-toxic lipid alcohols, thus inhibiting lipid peroxidation [31]. During ferroptosis, free extracellular ferrous ions enter cells via transferrin receptors and catalyze the conversion of lipid hydroperoxides into cytotoxic lipid free radicals (L–O–), leading to oxidative degradation of polyunsaturated fatty acids in cell membranes, and ultimately, cell death. Previous studies suggest that targeting ferroptosis and neuroinflammation represents a therapeutic strategy for depression [32]. Based on this rationale, we investigated the potential antidepressant effects mediated by TFAI through their modulation of ferroptosis and neuroinflammatory pathways. *In vitro* experiments using LPS-induced microglial cells revealed increased levels of intracellular iron ion and ROS, along with elevated MDA levels and reduced levels of SOD, GSH, and GSH-Px. Additionally, our results demonstrated reduced mRNA expression of the iron death-related gene *Gpx4*. Notably, TFAI treatment reversed these pathological changes. In conclusion, our findings demonstrated that TFAI exert antidepressant effects by inhibiting ferroptosis and neuroinflammation, partially through the regulation of *Gpx4*. These results offer new insights into the role of iron metabolism in the pathophysiology of depression.

The signaling of BDNF and its receptor TrkB is essential in the pathophysiology of depression and the mechanisms of antidepressant action [33]. BDNF also plays a key role in the survival, differentiation, and growth of peripheral and central neurons during development and adulthood [34]. BDNF is initially synthesized as a precursor protein (pre-pro BDNF) within the endoplasmic reticulum. Pro BDNF is converted to mature BDNF through intracellular and extracellular proteolytic cleavage. This mature BDNF form, upon binding to TrkB, activates the MAPK-CREB signaling cascade [35], which in turn enhances BDNF expression. MEK1 regulates the expression of ERK1/2, which, upon activation, phosphorylates downstream proteins such as CERB1 and BDNF that promote cell survival and exert neuroprotective effects [36]. The reduction of BDNF in the hippocampus is one of the causes of depression [37]. Inflammatory stimuli, such as LPS or pro-inflammatory cytokines, significantly reduce BDNF mRNA and protein expression *in vivo* [38]. Intraperitoneal injection of LPS in rats significantly reduced hippocampal BDNF mRNA expression. BDNF is an indispensable part of the pathophysiology of depression and the therapeutic mechanisms of antidepressant drugs. In the present study, we observed that BDNF levels in both hippocampal tissue and serum of a chronic CUMS mouse model were significantly reduced compared to those in the control. However, treatment with TFAI and fluoxetine restored BDNF levels in a dose-dependent manner. Our results indicate that TFAI significantly modulate the BDNF/TrkB/CREB signaling pathway, thereby alleviating depression symptoms and restoring physiological functions in CUMS-induced mice.

**Fig. 8:**
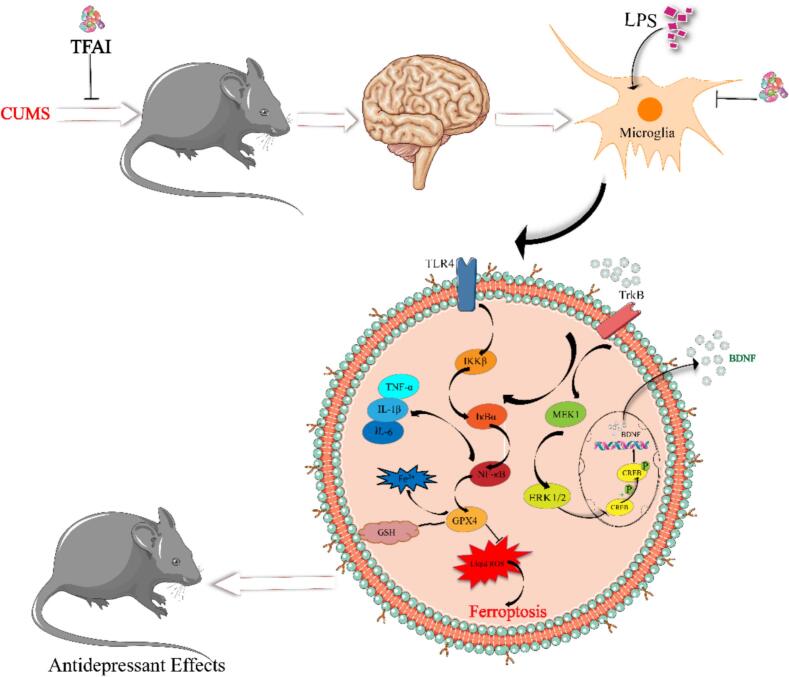
TFAI can modulate the expression of the BDNF/TrkB/CREB signaling pathway; TrkB:Tyrosine kinase receptor B; CREB1: cAMP responsive element binding protein 1/2; BDNF: brain derived neurotrophic factor.

In the present study, we demonstrated the antidepressant effects of TFAI and explored their potential mechanisms (Fig. 8). Our findings suggest that TFAI alleviate LPS-induced neuroinflammation and mitigate depression-like state and behaviors induced by CUMS. TFAI treatment significantly downregulated the gene expression of pro-inflammatory mediators in LPS-induced BV2 cells and reversed LPS-induced alterations in intracellular BDNF, SOD, MDA, GSH, ROS, and cytokine levels. Results of behavioral assessment indicated that CUMS-exposed mice exhibit a series of depression-like symptoms, including reduced motor ability, decreased sucrose preference, and increased immobility in TST and FST. Histopathological examination further confirmed specific neural damage in the hippocampus. Treatment with fluoxetine or TFAI significantly alleviated behavioral despair and partially restored hippocampal neuron integrity. Moreover, TFAI reversed the CUMS-induced decline in serum levels of 5-HT, DA, and BDNF. These findings collectively indicate that TFAI exert significant antidepressant effects, potentially through modulation of the TLR4/NF-κB/Gpx4 and the BDNF/TrkB/CREB signaling pathways in the hippocampal tissue.

In depression's pathophysiology, the TLR4/NF-κB/Gpx4 and BDNF/TrkB/CREB pathways form an interactive network via regulating inflammation, oxidative stress, and neuroplasticity [39]. TLR4 activates NF-κB through MyD88, inhibiting BDNF transcription, release, and TrkB expression via pro-inflammatory cytokines (e.g., IL-1β, TNF-α), and exacerbating oxidative stress via iNOS. NF-κB inhibition reverses BDNF downregulation in the nucleus accumbens, confirming inflammatory pathway regulation of neurotrophic signaling [40,41]. Gpx4, a key antioxidant enzyme, preserves neuronal membrane integrity and TrkB function by scavenging lipid peroxides; enhanced Gpx4 activity reduces ROS, inhibits NF-κB nuclear translocation and pro-inflammatory cytokine release, indirectly protecting BDNF expression [42]. Notably, BDNF/TrkB/CREB exerts reverse regulation: BDNF-TrkB activation of PI3K/Akt inhibits IKK to block NF-κB nuclear translocation [43], and TrkB signaling upregulates antioxidants like Gpx4 via Nrf2 [44]. This bidirectional mechanism highlights the “inflammation-oxidative stress-neuroplasticity impairment” cycle in depression. Targeted combined intervention on these pathways may offer precise depression treatments, with future research needed to explore cross-nodes like Nrf2 and ROS for new therapeutic targets..However, our study is not without limitations. Firstly, the pathway from NF-κB to Gpx4 is complex, and we only investigated a part of this pathway. Secondly, although we demonstrated the crucial role of Gpx4 in the action of TFAI in *in vitro* experiments, further research is needed to explore the potential mechanisms of iron death related to Gpx4 in depression.

## 5 Conclusions

In the present study, a DES system comprising ChCl and BDO in a 1:5 M ratio with 10 % water content was identified as the optimal solvent for extracting TFAI. The optimal extraction conditions determined by BBD were as follows: solid-to-liquid ratio, 32.97; extraction temperature, 68.74 °C; extraction time, 101 min; and ultrasonic power, 480.68 W. Under these optimal conditions, the total flavonoid yield reached 6.193412 mg/g. Further purification using D101 macroporous resin resulted in a TFAI dry powder with a yield of 2.2 % and a total flavonoid content of 27.72 %. The chemical composition of the extract was characterized using LC-MS/MS, and the content of marker compounds was quantified. The antidepressant activity of TFAI was evaluated through both *in vitro* and *in vivo* experiments. Treatment with the flavonoid extract significantly improved oxidative stress markers, including MDA, ROS, SOD, and GSH, in LPS-induced BV2 microglial cells. RT-PCR analyses revealed that TFAI significantly downregulated mRNA expression levels of *TLR4*, *IKKβ*, *NF-κB*, *IL-1β*, and *TNF-α* compared to the control. *In vivo*, TFAI significantly reduced despair behaviors in depressed mice subjected to CUMS and partially restored hippocampal neuronal integrity. Additionally, TFAI reversed CUMS-induced reductions in 5-HT, DA, and BDNF levels. RT-PCR and immunohistochemistry results confirmed that the antidepressant effects of TFAI are mediated, at least in part, through activation of the BDNF/TrkB/CREB signaling pathway.

This study successfully optimized the TFAI extraction process using a DES-based, ultrasound-assisted extraction strategy, highlighting the application of sonochemistry in green extraction and bioactive compound processing. Moreover, by elucidating the dual-action mechanism of TFAI through the modulation of inflammatory and neurotrophic pathways, we expanded their potential as both dietary supplements and therapeutic agents—extending their applications beyond traditional cardiovascular protection to include the prevention and treatment of neuropsychiatric disorders. This study fills a critical gap in the neuropharmacological research of *A. italica* Retz.

## CRediT authorship contribution statement

**Bingchen Han:** Writing – review & editing, Writing – original draft, Methodology, Investigation, Formal analysis, Data curation, Conceptualization. **Qindan Cui:** Writing – original draft, Software, Methodology, Investigation, Formal analysis, Data curation. **Zhiliang Ma:** Investigation. **Leiling Shi:** Investigation, Funding acquisition. **Yu Sun:** Investigation. **Jiawei Dai:** Investigation, Formal analysis, Data curation. **Jun Deng:** Visualization, Validation, Methodology, Formal analysis, Data curation. **Han Cheng:** Writing – review & editing, Methodology, Conceptualization. **Jun Li:** Writing – review & editing, Funding acquisition, Conceptualization. **Yuebin Ge:** Supervision. **Xianju Huang:** Writing – review & editing, Supervision, Methodology, Investigation, Funding acquisition.

## Funding

This study was supported by National Key R&D Program of China (2024YFC3506700 & 2024YFC3506704),Hubei Science and Technology Innovation Base (platform) (2024CFC003), Key Research and Development Special Project of Hubei Provincial Science and Technology Plan (2024BBB033), International cooperation projects in Hubei province (2022EHB053), the Fund for Academic Innovation Teams of South-Central Minzu University (XTZ24025) and the Fundamental Research Funds for the Central Universities of South-Central Minzu University (3212025yjshq033), the Science and Technology Development Fund of the Macau SAR (Ref no.: 001/2023/ALC) and the Open Research Project of the Macao Centre for Research and Development in Chinese Medicine (No. MCRDCM-OP2511).

## Declaration of competing interest

The authors declare that they have no known competing financial interests or personal relationships that could have appeared to influence the work reported in this paper.
