# Supplementary material for: Ultrasound-assisted deep eutectic solvent extraction of bioactive flavonoids from Anchusa italica retz.: optimization, characterization, and evaluation of antidepressant potential − targeting oxidative stress and neurotrophic pathways
Source: Ultrason Sonochem. 2025 Oct 4;122:107598. doi: 10.1016/j.ultsonch.2025.107598 (PMC12538459; doi:10.1016/j.ultsonch.2025.107598)
Supplement: Supplementary Data 1 [file mmc1.docx]

**Ultrasound-Assisted Deep Eutectic Solvent Extraction of Bioactive Flavonoids from *Anchusa italica* Retz.: Optimization, Characterization, and Evaluation of Antidepressant Potential - Targeting Oxidative Stress and Neurotrophic Pathways**

Bingchen Han^1^^,3†^; Qindan Cui^1, 4†^; Zhiliang Ma^5^; Leiling Shi^6^; Yu Sun^6^; Jiawei Dai^1^; Jun Deng^1^; Han Cheng^1,2^; Jun Li^1,2^; Yuebin Ge^1,2^; Xianju Huang^1,2*^^[[1]](#footnote-1)^

*^1^* *Hubei International Science and Technology Cooperation Base (SH2311), South-Central Minzu University, Wuhan 430079, China*

*^2^* *School of Pharmaceutical Sciences, South-Central Minzu University, Wuhan 430079, China*

*^3^ College of life Sciences, South-Central Minzu University, Wuhan 430079, China*

*^4^Department of Pharmacy, Sichuan Provincial People's Hospital East Sichuan & Dazhou First People's Hospital, Dazhou, 635000, China*

*^5^ Qinghai Tibetan Medicine Research Institute，Qinghai Xining 810016,China*

^6^ *Xinjiang Institute of Chinese and Ethnic Medicine, Urumqi 830002, China*

**Table.S.1** The stressors of a CUMS procedure.

| Serial number | HBA/HBD | Mole ratio | References |
| --- | --- | --- | --- |
| DES-1 | ChCl/Urea | 1:2 | [1] |
| DES-2 | ChCl/Glu | 1:1 | [2] |
| DES-3 | ChCl/LA | 1:2 | [3] |
| DES-4 | ChCl/OA | 1:1 | [4] |
| DES-5 | ChCl/MA | 1:1 | [5] |
| DES-6 | ChCl/BDO | 1:5 | [6] |
| DES-7 | ChCl/Gly | 1:2 | [7] |
| DES-8 | ChCl/EG | 1:1 | [8] |

**[**1] M. Huang, Y. Fu, P. Wei, Y. Li, Ultrasound-assisted deep eutectic solvent extraction of flavonoids from pericarp of cultivated ‘Qi-nan’ agarwood: identification, optimization, and antioxidant activity, Microchemical Journal, 212 (2025) 113303.

[2] F. Ianni, S. Scandar, L. Mangiapelo, F. Blasi, M.C. Marcotullio, L. Cossignani, NADES-Assisted Extraction of Polyphenols from Coriander Seeds: A Systematic Optimization Study, in: Antioxidants, 2023.

[3] A. Olfat, T. Mostaghim, S. Shahriari, M. Salehifar, Extraction of bioactive compounds of Hypnea flagelliformis by ultrasound-assisted extraction coupled with natural deep eutectic solvent and enzyme inhibitory activity, Algal Research, 78 (2024) 103388.

[4] H. Huang, P. Ying, Y. Wang, Q. Wu, L. Wang, X. Fu, Temperature-dependent convection induced incremental extraction of anthocyanins from Melastoma dodecandrum Lour. Based on recyclable natural deep eutectic system, Food Chemistry, 484 (2025) 144331.

[5] A.P. Abbott, D. Boothby, G. Capper, D.L. Davies, R.K. Rasheed, Deep Eutectic Solvents Formed between Choline Chloride and Carboxylic Acids:  Versatile Alternatives to Ionic Liquids, Journal of the American Chemical Society, 126 (2004) 9142-9147.

[6] Q. Cao, J. Li, Y. Xia, W. Li, S. Luo, C. Ma, S. Liu, Green Extraction of Six Phenolic Compounds from Rattan (Calamoideae faberii) with Deep Eutectic Solvent by Homogenate-Assisted Vacuum-Cavitation Method, in: Molecules, 2019.

[7] G. Muhammad, J. Wang, W. Xiong, Y. Lv, S. Zhang, A. Zhao, P. Jahanbakhsh-Bonab, A. Solovchenko, J. Xu, M.A. Alam, Polyol based deep eutectic solvent-assisted pretreatment for enhanced lutein extraction from Chlorella pyrenoidosa, Journal of Molecular Liquids, 368 (2022) 120775.

[8] G. Muhammad, P. Jahanbakhsh-Bonab, W. Xiong, Y. Lv, S. Zhang, A. Zhao, J.J. Sardroodi, J. Xu, M.A. Alam, Mechanism of deep eutectic solvent-mediated microalgal biomass disintegration for enhanced lutein extraction, Industrial Crops and Products, 209 (2024) 117940.

**Table.S.2** The stressors of a CUMS procedure.

| Stressor | Duration |
| --- | --- |
| food and water deprivation | 24h |
| Food deprivation | 12h |
| Water deprivation | 12h |
| Illumination overnight | 12h |
| Tilted cage for 45° | 12h |
| Wet cage | 12h |
| crowding | 12h |
| empty cage | 12h |
| Physical restrain | 2h |
| tail suspension | 10min |
| Clip tail | 10min |
| Force swimming in cold water | 6min |

**Table.S.3** Primer sequences used in the qRT-PCR

| **Gene name** |  | **Primer sequences** |
| --- | --- | --- |
| Trk B | Forward: | 5’-ATCACCAACAGTCAGCTCAAGC-3’ |
|  | Reverse: | 5’- TTCAGCGTCTTCACAGCCAC -3’ |
| CREB | Forward: | 5’-AGCCGGGTACTACCATTC-3’ |
|  | Reverse: | 5’-GCTGCTTCCCTGTTCTTC-3’ |
| BDNF | Forward: | 5’-TGGCTGACACTTTTGAGCACGTC -3’ |
|  | Reverse: | 5’-GCTCCAAAGGCACTTGACTGCTGA-3’ |
| NLRP3 | Forward: | 5’-TAAGAACTGTCATAGGGTCAAAACG-3’ |
|  | Reverse: | 5’-GTCTGGAAGAACAGGCAACATG-3’ |
| HSP90 | Forward: | 5’-GATATGGCTATTGCTACTGGTGGTGC-3’ |
|  | Reverse: | 5’-CCTAAGTCATGAGCTTGAACATCTTC-3’ |
| GAPDH | Forward: | 5’-CACTCACGGCAAATTCAACGGCAC-3’ |
|  | Reverse: | 5’-GACTCCACGACATACTCAGCAC-3’ |
| TLR4 | Forward: | 5’-TTCAGAACTTCAGTGGCTGGATTTA-3’ |
|  | Reverse: | 5’-GTCTCCACAGCCACCAGATTCTC-3’ |
| GPX4 | Forward: | 5’-TGTGCATCCCGCGATGATT-3’ |
|  | Reverse: | 5’-CCCTGTACTTATCCAGGCAGA-3’ |
| NF-κB | Forward: | 5’- TGCGATTCCGCTATAAATGCG-3’ |
|  | Reverse: | 5’- ACAAGTTCATGTGGATGAGGC-3’ |
| TNF-α | Forward: | 5’- TTGACCTCAGCGCTGAGTTG-3’ |
|  | Reverse: | 5’- CCTGTAGCCCACGTCGTAGC-3’ |
| IL-1β | Forward: | 5’-TGCCACCTTTTGACAGTGAT-3’ |
|  | Reverse: | 5’-GTGCTGCTGCGAGATTTGAA-3’ |
| IL-6 | Forward: | 5’- AGCTATGAACTCCTTCTCCAC -3’ |
|  | Reverse: | 5’- GTTTGTCAATTCGTTCTGAAG -3’ |
| IKKβ | Forward: | 5’- TAGTAGAGCGGATGATGGCA-3’ |
|  | Reverse: | 5’- CTTCT CCCTGAGTCTTCGGTA-3’ |

**Table.S.4** ANOVA for Response Surface Quadratic Model

|  | Sum of |  | Mean | F | p-value |  |
| --- | --- | --- | --- | --- | --- | --- |
| Source | Squares | df | Square | Value | Prob > F |  |
| Model | 0.897 | 14 | 0.064071 | 24.76781 | < 0.0001 | significant |
| A-Liquid to material ratio | 0.384685 | 1 | 0.384685 | 148.7059 | < 0.0001 |  |
| B-Temperature | 0.02769 | 1 | 0.02769 | 10.70403 | 0.0056 |  |
| C-Time | 0.017868 | 1 | 0.017868 | 6.907162 | 0.0199 |  |
| D-Ultrasonic power | 0.014645 | 1 | 0.014645 | 5.661077 | 0.0321 |  |
| AB | 0.000275 | 1 | 0.000275 | 0.106208 | 0.7493 |  |
| AC | 0.018527 | 1 | 0.018527 | 7.162067 | 0.0181 |  |
| AD | 0.02391 | 1 | 0.02391 | 9.242953 | 0.0088 |  |
| BC | 0.016596 | 1 | 0.016596 | 6.415397 | 0.0239 |  |
| BD | 3.98E-06 | 1 | 3.98E-06 | 0.001539 | 0.9693 |  |
| CD | 0.000169 | 1 | 0.000169 | 0.06534 | 0.8020 |  |
| A^2 | 0.372523 | 1 | 0.372523 | 144.0048 | < 0.0001 |  |
| B^2 | 0.008075 | 1 | 0.008075 | 3.121684 | 0.0990 |  |
| C^2 | 0.038011 | 1 | 0.038011 | 14.69387 | 0.0018 |  |
| D^2 | 0.052004 | 1 | 0.052004 | 20.10312 | 0.0005 |  |
| Residual | 0.036216 | 14 | 0.002587 |  |  |  |
| Lack of Fit | 0.028281 | 10 | 0.002828 | 1.425482 | 0.3915 | not significant |
| Pure Error | 0.007936 | 4 | 0.001984 |  |  |  |
| Cor Total | 0.933216 | 28 |  |  |  |  |

**Table.S.5** Chemical Composition Identification Form of TFAI

| **NO.** | **T_R_/(min)** | **Formula** | **Calc. MW** | **Error (ppm)** | **mzCloud Best Match** | **ESI** | **MS^2^** | **Potential compounds** |
| --- | --- | --- | --- | --- | --- | --- | --- | --- |
| 1 | 0.903 | C_6_H_13_NO_5_ | 179.0798 | -2.29 | 84.6 | H^+^ | 163.2、145.1 | D-Glucosamine |
| 2 | 1.02 | C_10_H_13_N_5_O_4_ | 267.0974 | -2.27 | 88.5 | H^+^ | 251.1、237.1 | Adenosine |
| 3 | 1.249 | C_6_H_6_N_2_O | 122.0485 | -0.52 | 83.5 | H^+^ | 123.05、106.02 | Nicotinamide |
| 4 | 10.534 | C_15_H_10_O_7_ | 302.0432 | 1.91 | 90.4 | H^+^ | 285.04、257.04 | Quercetin |
| 5 | 10.536 | C_27_H_30_O_16_ | 610.1548 | 2.27 | 87.3 | H^+^ | 465.01、303.05 | Rutin |
| 6 | 10.54 | C_21_H_20_O_12_ | 464.0966 | -2.63 | 88.8 | H^+^ | 353.09、303.04 | Quercetin-3β-D-glucoside |
| 7 | 10.542 | C_21_H_20_O_11_ | 448.1014 | 1.93 | 83.7 | H^+^ | 303.04、129.05 | Quercitrin |
| 8 | 11.734 | C_15_H_10_O_6_ | 286.0483 | 1.95 | 91.5 | H^+^ | 259、213、185 | Kaempferol |
| 9 | 11.735 | C_21_H_20_O_11_ | 448.1014 | -2.3 | 82.9 | H^-^ | 327.04、286.04 | Astragalin |
| 10 | 11.737 | C_27_H_30_O_15_ | 594.1598 | 2.3 | 83.2 | H^+^ | 463.12、288.05 | Nictoflorin |
| 11 | 11.981 | C_9_H_8_O_4_ | 180.0426 | 1.84 | 87.1 | H^-^ | 179.2、135.0 | Caffeic acid |
| 12 | 12.075 | C_16_H_12_O_7_ | 316.0591 | 2.42 | 87.7 | H^+^ | 303.04、283.2 | Isorhamnetin |
| 13 | 23.966 | C_18_H_24_O_3_ | 288.1731 | -1.74 | 81.6 | H^+^ | 159.07 | Estriol |
| 14 | 27.359 | C_21_H_20_O_6_ | 368.1267 | -1.23 | 83.2 | H^+^ | 285.1、259.1 | Curcumin |
| 15 | 30.131 | C_15_H_22_O_2_ | 234.1625 | -1.97 | 92.3 | H^+^ | 179.1、163.07 | 3,5-di-tert-Butyl-4-hydroxybenzaldehyde |
| 16 | 30.608 | C_18_H_30_O_2_ | 278.2251 | 1.92 | 86.6 | H^-^ | 259.2、277.2 | pinolenic acid |
| 17 | 43.54 | C_24_H_38_O_4_ | 390.2781 | -1.44 | 87.9 | H^+^ | 279.15、167.03 | Bis(2-ethylhexyl) phthalate |

**Table.S.6** the standard curves, linear ranges, and sample contents of the four constituents

| **Compound** | **The standard curve** | **Linear range(µg/mL)** | **R^2^** | **Sample (mg/g)** |
| --- | --- | --- | --- | --- |
| Astragalin | y = 1310.5x + 4.0463 | 0.128-0.96 | 0.9998 | 14.25 |
| Isorhamnetin | y = 1909.6x - 2.8616 | 0.13-1.95 | 0.9992 | 3.87 |
| Rutin | y=771.9x - 5.5922 | 0.144-1.08 | 0.9957 | 11.30 |
| Nicotiflorin | y=569.72x + 7.3659 | 0.154-1.54 | 0.9996 | 14.79 |


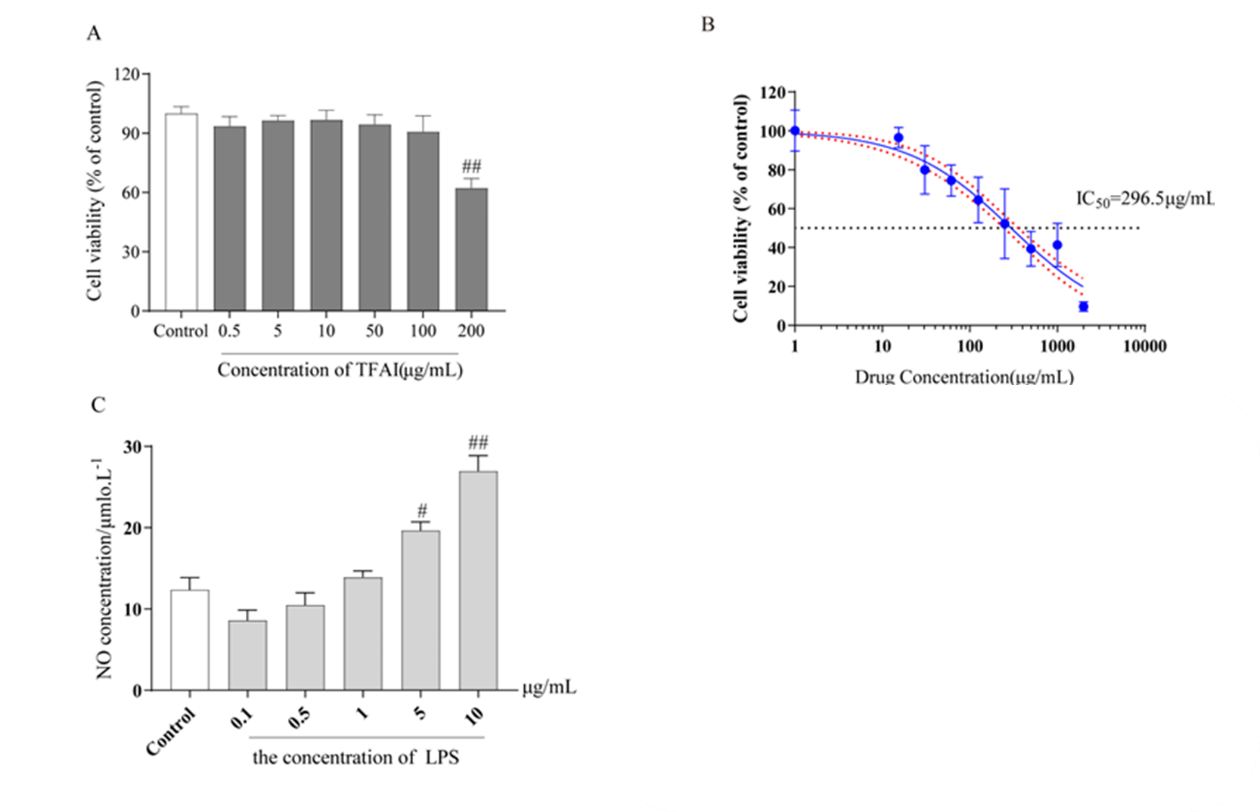


**Fig.S.1** (A) quantitative analysis of TFAI on BV2 cell viability; (B) IC50 of TFAI on BV2 cells; (C) Effect of different concentrations of LPS on NO content in the supernatant of BV2 cells;


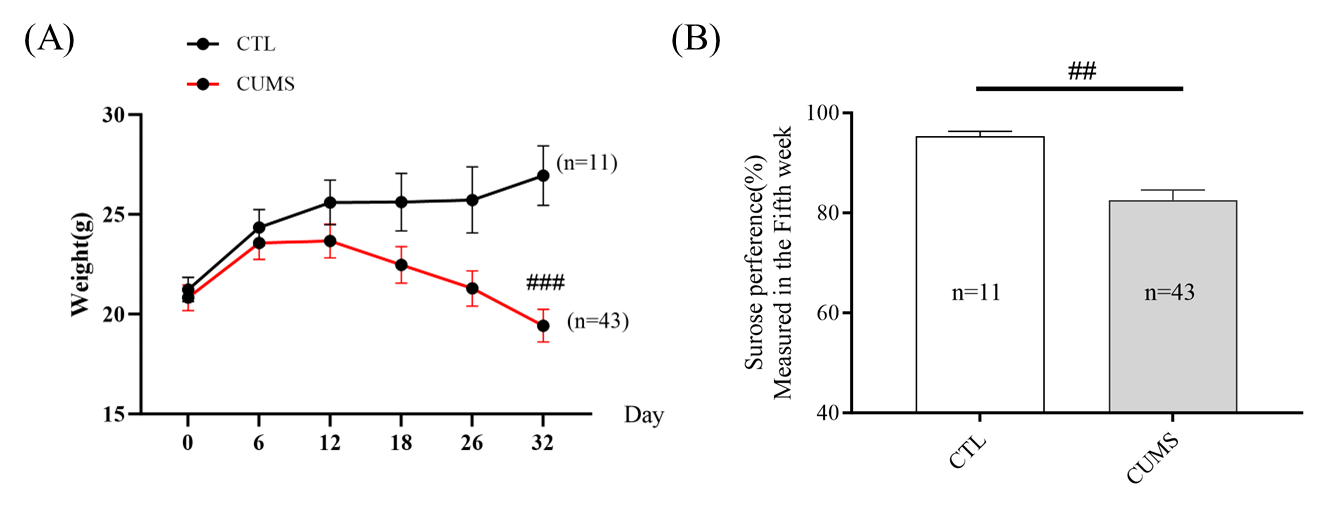


**Fig.S.2** (A) Body weight changes in mice after 5 weeks CUMS modeling; (B) Sucrose preference test in mice after 5 weeks CUMS model;


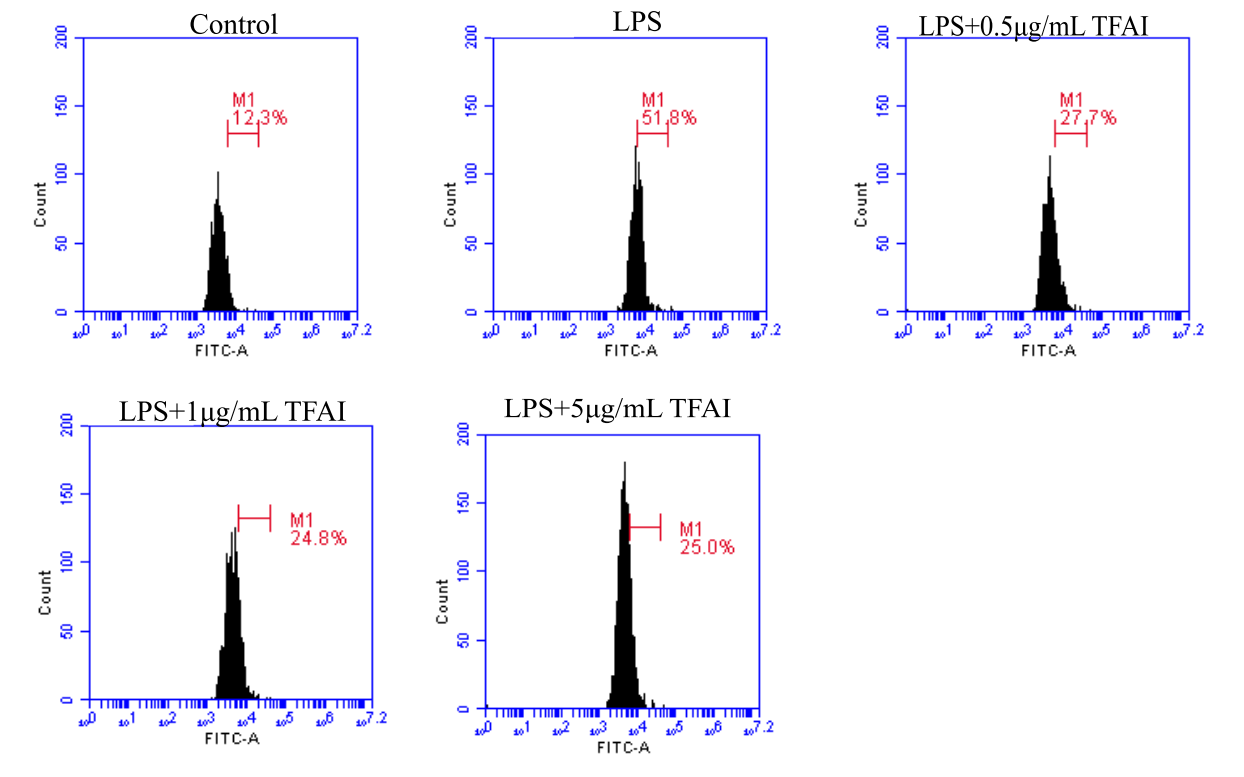


**Fig.S.3** Fluorescence intensity analysis of ROS

1. †These authors contributed equally to this work.

   * Correspoonding Author: Xianju Huang, Ph. D., Professor. South-Central Minzu University, 182 Minyuan Road, Wuhan, P.R. China, 430074. Tel: 0086-27-67841196; E-mail: (X-J Huang) [xianju@mail.scuec.edu.cn；](mailto:xianju@mail.scuec.edu.cn；) [↑](#footnote-ref-1)
